# Increased Expression of Complement Regulators CD55 and CD59 on Peripheral Blood Cells in Patients with EAHEC O104:H4 Infection

**DOI:** 10.1371/journal.pone.0074880

**Published:** 2013-09-23

**Authors:** Werner Dammermann, Pim Schipper, Sebastian Ullrich, Katharina Fraedrich, Julian Schulze zur Wiesch, Thorben Fründt, Gisa Tiegs, Ansgar Lohse, Stefan Lüth

**Affiliations:** 1 Department of Medicine, University Medical Center Eppendorf, Hamburg, Germany; 2 Department of Anatomy and Experimental Morphology, University Medical Center Eppendorf, Hamburg, Germany; 3 Institute of Experimental Immunology and Hepatology, University Medical Center Eppendorf, Hamburg, Germany; The Hospital for Sick Children and The University of Toronto, Canada

## Abstract

**Background:**

An outbreak of Shiga Toxin 2 (Stx-2) producing enterohemorrhagic and enteroaggregative *E.coli* (EAHEC) O104H4 infection in May 2011 caused enterocolitis and an unprecedented high 22% rate of hemolytic uremic syndrome (HUS). The monoclonal anti-C5 antibody Eculizumab (ECU) has been used experimentally in EAHEC patients with HUS but treatment efficacy is uncertain. ECU can effectively prevent hemolysis in paroxysmal nocturnal hemoglobinuria (PNH) caused by a lack of complement-regulating CD55 and CD59 on blood cells. We hypothesized a low expression of CD55 and CD59, as seen in PNH, might correlate with HUS development in EAHEC patients.

**Methods:**

76 EAHEC patients (34 only gastrointestinal symptoms [GI], 23: HUS, 19: HUS and neurological symptoms [HUS/N]) and 12 healthy controls (HC) were tested for the expression of CD55 and CD59 on erythrocytes and leukocytes retrospectively. Additionally, the effect of Stx-2 on CD55 and CD59 expression on erythrocytes and leukocytes was studied *ex vivo*.

**Results:**

CD55 expression on erythrocytes was similar in all patient groups and HC while CD59 showed a significantly higher expression in HUS and HUS/N patients compared to HC and the GI group. CD55 and CD59 expression on leukocytes and their subsets was significantly higher in all patient groups compared to HC regardless of treatment type. However, CD59 expression on erythrocytes was significantly higher in HUS and HUS/N patients treated combined with plasma separation (PS) and ECU compared to HC. Adding Stx-2 *ex vivo* had no effect on CD55 and CD59 expression on leukocytes from HC or patients.

**Conclusion:**

HUS evolved independently from CD55 and CD59 expression on peripheral blood cells in EAHEC O104:H4 infected patients. Our data do not support a role for CD55 and CD59 in HUS development during EAHEC O104:H4 infection and point to a different mechanism within the complement system for HUS development in EAHEC patients.

## Introduction

The outbreak of infection with enterohemorrhagic and enteroaggregative *E.coli* (EAHEC) serotype O104:H4 in northern Germany which affected 3842 patients until August 2011 was characterized by an unusually high rate of hemolytic uremic syndrome HUS (22% vs. 5–10% in other EHEC outbreaks) and neurological complications [1]–[4].

The production of Shiga toxin 2 (Stx-2) of the new enteroaggregative serotype O104:H4 was thought to be responsible for the high rate of complications and the death of 53 patients [2], [3], [5]. So far, most prevalent treatment of patients developing HUS is plasma separation (PS). A positive report on 3 EHEC infected children suffering from severe Shiga toxin associated HUS led to the empirical use of eculizumab (ECU) [6]. ECU is a monoclonal anti-C5 antibody which inhibits C5 activation, thus blocks the terminal pathway of the complement system irrespective of lacking CD55 and CD59 on peripheral blood cells and is most effective in paroxysmal nocturnal hemoglobinuria (PNH) [1], [4]. Due to a high rate of spontaneous resolution of HUS in EAHEC infected patients, the effect of ECU in HUS due to EAHEC infection is difficult to determine. Furthermore, the positive report on 3 EHEC infected children is debatable, since the platelet and LDH levels were starting to recover at the time of antibody treatment [6]. So there remains uncertainty whether treatment was efficacious or coincident with natural recovery.

PNH is a hemolytic disorder, which is characterized by a lack of complement regulators CD55 and CD59 on erythrocytes and leukocytes and leads to hemolytic anemia. CD55 and CD59 normally prevent complement-mediated lysis of host cells [7]: CD55 binds C3b and C4b and decay dissociates Bb and C2a from the C3bBb and C4bC2a convertases. CD59 binds to the terminal C5b-8 and C5b-9 intermediate complexes and prevents the full assembly of the membrane attack complex.

We hypothesized that low expression of CD55 and CD59 either constitutively, as observed in PNH, or after Stx-2 challenge contributes to the unprecedented high rate of HUS in EAHEC O104:H4 infected patients. Thus, low CD55 and CD59 expression in EAHEC infected patients might be helpful to identify patients at risk of HUS development and to recognize patients with a benefit from ECU treatment.

## Methods

### Patient Selection

Patients treated for infection with E. Coli O104:H4 in the University Medical Center Hamburg-Eppendorf were enrolled in the study retrospectively for which all patients gave written consent and which was approved by the *Ethics Committee of the Hamburg Chamber of Physicians*. (# PV3826).

Subjects were enrolled and samples collected during follow up 3 months after release from hospital based on previous presence of: abdominal pain, bloody diarrhea (≥3 stools/24 h) at time of admission and a positive identification of E. Coli O104:H4 in stool culture. Patients were stratified retrospectively according to their clinical course into 3 groups with either severe gastrointestinal symptoms (GI, n = 34), HUS without neurological symptoms (HUS, n = 23) and HUS with neurological symptoms (HUS/N, n = 19). The HUS and HUS/N groups were subdivided based on the treatment they received (plasma separation alone or plasma separation and eculizumab (Soliris®)). Treatment followed the same protocols for all patients. The selected patients are highly representative and depict the distribution in the total pool of patients with EAHEC infection who were admitted and treated at our hospital (GI: 44%, HUS: 30%, HUS/N: 26%). Healthy subjects were selected out of a group of volunteers which were representative to the patients group.

### Flow Cytometry Analysis

#### Erythrocyte sample preparation

Whole blood samples were freshly drawn in EDTA blood sample tube and were refrigerated at 4°C until sample preparations started within a maximum of 24 hours after blood collection. 1 µL of whole blood was added to 100 µL of F-solution (98% v/v PBS, 1% v/v FBS (Lonza, catalogue nr. DE14–802F) and 1% of 9% w/v NaN_3_ solution, stored at 4°C) in 5 mL flow cytometry tubes (Sarstedt, catalogue no. 55.1579) and then vortexed. 5 µL of FC-blocking reagent (Biolegend, catalogue no. 422302) was subsequently added to the tubes, which were then vortexed and incubated for 10 minutes. Isotype-controls and antibodies (see table S1) were then added to the tubes. When all isotype-controls or antibodies were added, the tubes were then vortexed and incubated for 30 minutes.

Next, the erythrocytes samples were washed from platelets and cell debris. Tubes were centrifuged in an Eppendorf 5810R centrifuge at 200 RCF for 5 minutes. The supernatant was removed and 2 mL of F-solution was added and the tubes were then vortexed. This procedure was repeated once with 0.5 mL of F-solution. Thereafter the tubes were put on ice and were protected from light until analysis at the flow cytometer, within 5 to 15 minutes.

#### Leukocyte sample preparation

50 µL of whole blood was put into 5 mL flow cytometry tubes. 5 µL of FC-blocking reagent was subsequently added to the tubes, which were then vortexed and incubated for 10 minutes. Isotype-controls and antibodies (see table S1) were added to the tubes. When all isotype-controls or antibodies were added, the tubes were vortexed and incubated for 30 minutes.

Subsequently the erythrocytes were lysed by adding 450 µL of FACS lysing solution (BD Biosciences, catalogue nr. 349202) and incubating the tubes for 20 minutes. Thereafter the tubes were put on ice and protected from light until analysis at the flow cytometer, within 5 to 15 minutes.

All incubation mentioned was done by incubating the tubes protected from light at room temperature (15–25°C). Samples were stored on ice and protected from light until analysis. The tube was then vortexed and put in to the flow cytometer for analysis.

### Ex Vivo Exposure of Whole Blood to Stx-2

Immediately after blood collection blood was incubated with 0.9% NaCl as a negative control and Shiga toxin 2 (List Biological Laboratories, Inc. USA) in two concentrations, 10 ng/mL (Stx-2 condition 1) and 0.1 ng/mL (Stx-2 condition 2). The samples were then co-incubated for 24 hours at 37°C, 5% (v/v) CO_2_) before preparation and analysis.

### Flow Cytometer

The samples were analyzed using a BD FACSCanto II flow cytometer. Raw data was extracted using the BD FACSDiva Software Version 6.1. The flow cytometry data was analyzed using FCS Express 4 Flow Cytometry Professional Standalone Research Edition (DeNovo Software, version number 4.01.0018).

### Data Analysis

#### Statistical analysis

ANOVA was performed on all flow cytometric data except for the data resulting from the Stx-2 incubation experiments where the Student unpaired T-test was performed due to a small population size.

Pearson-Bravais correlation was performed on all flow cytometric data and blood parameter data.

## Results

### Higher Expression of CD59 on Erythrocytes and Leukocytes of All Patients Previously Infected with EAHEC O104:H4 Irrespective of the Clinical Course

Seventy six patients were consecutively selected out of a group of 182 fully recovered patients previously infected with EAHEC O104:H4 (all patients initially presented with bloody diarrhea) during follow up three months after discharge from the hospital. Patients were grouped according to their previous clinical course into 3 groups with either severe gastrointestinal symptoms (GI, n = 34), HUS without neurological symptoms (HUS, n = 23) and HUS with neurological symptoms (HUS/N, n = 19) as depicted in table 1 and 2. The primary objective was to look for constitutive expression levels of CD55 and CD59 on erythrocytes and leukocytes as well as different leucocyte subsets via flow cytometry within the three groups of former patients. Results from all patient groups were compared to those drawn from twelve healthy controls (HC) and are shown in an additional figure in more detail (see figure S1).

**Table 1:** Characteristics of all subjects included in the study.

Characteristic						
			HC^c^	GI^c^	HUS^c^	HUS/N^c^
			(n = 12)	(n = 34)	(n = 23)	(n = 19)
**Age (yr)** a			27.4±3.8	44.9±16.8	36.2±14.8	37.0±17.1
**Gender**						
	Female		6 (50.0%)	20 (58.8%)	16 (69.6%)	14 (73.7%)
**Treatment plasma seperation** b						
	Patients		−	−	9	3
	Age (yr)^a^		−	−	33.0±12.3	54.3±10.6
	Gender		−	−		
		Female	−	−	7 (77.8%)	2 (66.7%)
**Treatment plasma seperation+eculizumab** b						
	Patients		−	−	14	16
	Age (yr)a		−	−	38.3±15.9	33.8±16.1
	Gender		−	−		
		Female	−	−	9 (64.3%)	12 (75.0%)
**In-vitro Stx-2 exposure**						
	Patients		4	4	4	4
	Age (yr)^a^		27.0±7.1	32.0±4.2	27.5±2.1	38.0±18.8
	Gender					
		Female	2 (50%)	2 (50%)	2 (50%)	3 (75%)

a The data are shown as means ± standard deviations.

b The groups HC and GI were neither treated with plasma seperation or eculizumab.

c HC = healthy controls, GI = gastrointestinal symptoms, HUS = hemolytic uremic syndrome, HUS/N = HUS and neurological symptoms.

**Table 2:** Blood parameters for hemoglobin, thrombocytes, urea and creatinine of all patients included in the study.

Characteristic			
	GI^b^	HUS^b^	HUS/N^b^
	n = 34	n = 23	n = 19
hemoglobin^a^ [g/dL]	13.54±1.23	12.99±1.37	11.91±1.67
thrombocytes^a^[10^9^/L]	254.68±48.73	266.91±46.93	248.42±69.25
urea^a^ [mg/dL]	13.79±4.29	12.91±3.81	20.05±10.76
creatinine^a^ [mg/dL]	0.88±0.16	0.93±0.31	1.33±0.74

a The data are shown as means ± standard deviations.

b HC = healthy controls, GI = gastrointestinal symptoms, HUS = hemolytic uremic syndrome, HUS/N = HUS and neurological symptoms reference levels: hemoglobin: 14–17.5 g/dL, thrombocytes: 150–400 10^9^/L, urea: 8–26 mg/dL, creatinine: 0.6–1.3 mg/dL.

**Figure 1:**
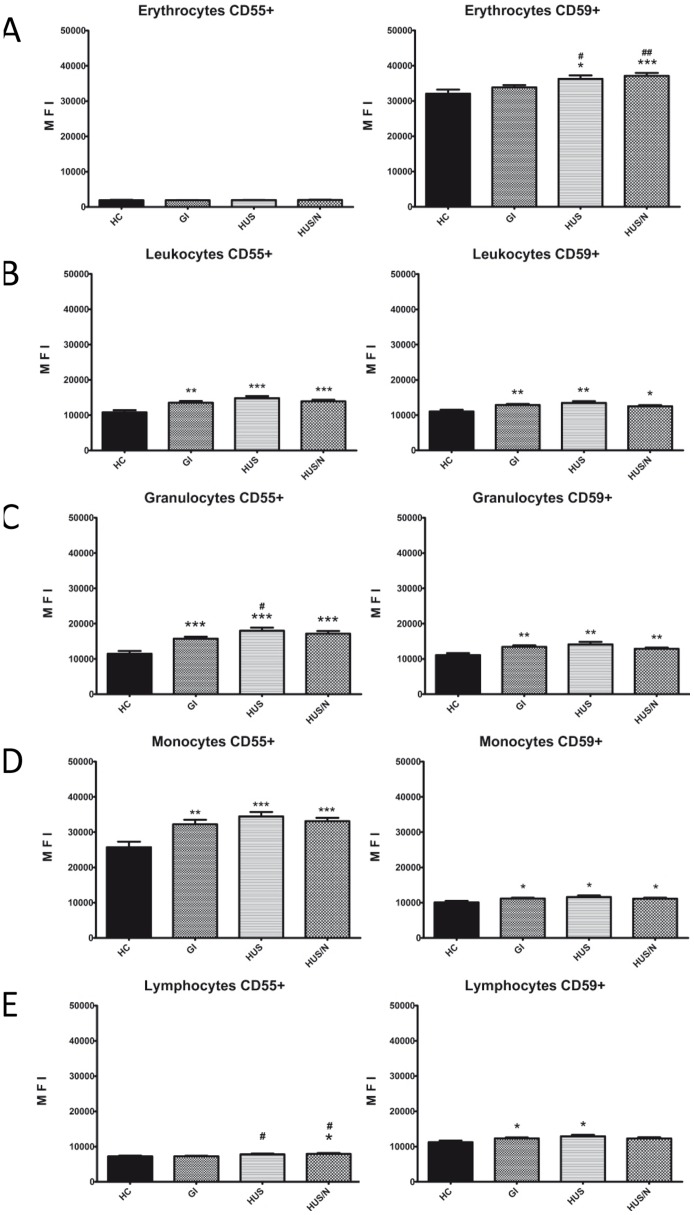
CD55 and CD59 expression are increased on erythrocytes and leukocytes of former EAHEC-infected patients. Erythrocytes and leukocytes were incubated with CD45-, CD55- and CD59-specific antibodies and analyzed via flow cytometry. The CD45 marker was used to exclude or include the leukocyte population whereas the leukocyte subsets were distinguished via FSC/SSC-plot. Patients were grouped according to their clinical course into 3 groups with either severe gastrointestinal symptoms (GI, n = 34), HUS without neurological symptoms (HUS, n = 23) and HUS with neurological symptoms only (HUS/N, n = 19). Healthy controls were also screened (HC, n = 12). A Erythrocytes, B Leukocytes, C Granulocytes, D Monocytes, E Lymphocytes. All values are given as mean fluorescence intensity (MFI) ± S.D. ANOVA, following symbols are used to pinpoint significant differences: vs. HC *, vs. GI #. One symbol equals 0.05, two symbols 0.01, three symbols 0.001.

We found no differences in CD55 expression on erythrocytes between all of the three patient groups and of the healthy controls (Fig. 1 A). The HUS and HUS/N groups showed a significantly higher expression of CD59 when compared to HC (Fig. 1 A; 36282 and 37156 vs. 32068, p = 0.0140 and p = 0.0009, respectively) and to the GI group (36282 and 37156 vs. 33852, p = 0.0397 and p = 0.0026, respectively).

CD55 and CD59 expression on leukocytes and their subsets (granulocytes, monocytes and lymphocytes) were significantly higher in all patient groups (GI, HUS and HUS/N) compared to HC (Fig. 1 B; 13558, 14849 and 13941 vs. 10805, p = 0.0022, p<0.0001 and p = 0.0001, respectively for CD55; 12870, 13451 and 12,514 vs. 11039, p = 0.0085, p = 0.0082 and p = 0.0174, respectively for CD59). However, no differences were found between the three patient groups.

Granulocytes also showed a higher expression for both CD55 and CD59 within all patient groups when compared to HC (Fig. 1 C; 15760, 17986 and 17180 vs. 11465, p = 0.0002, p<0.0001 and p<0.0001, respectively for CD55; 13426, 14156 and 12862 vs. 11091, p = 0.0057, p = 0.0083 and p = 0.0081, respectively for CD59). The CD55 expression was also significantly higher in the HUS group compared to the GI group (17986 vs. 15760 p = 0.0307).

Similar to granulocytes, monocytes had a significantly higher expression for both CD55 and CD59 within all 3 patient groups compared to HC (Fig. 1 D; 32226, 34474 and 33123 vs. 25754, p = 0.0088, p = 0.0002 and p = 0.0002, respectively for CD55; 11188, 11600 and 11165 vs. 10078, p = 0.0217, p = 0.0314 and p = 0.0245, respectively for CD59).

On lymphocytes CD55 expression was significantly higher in the HUS and HUS/N groups compared to HC (Fig. 1 E; 7824 and 7944 vs. 7246, p = 0.0638 and p = 0.0488) and the GI group (7824 and 7944 vs. 7252, p = 0.0233 and p = 0.0107 respectively). We observed no difference between the GI and HC group.

CD59 expression on lymphocytes was also significantly higher in the GI and HUS groups compared to HC (Fig. 1 E; 12361 and 12922 vs. 11225, p = 0.0416 and p = 0.0277 respectively). Whereas only a trend towards a significant difference was found when comparing the HUS/N group with HC (12290 vs. 11225, p = 0.0966).

Blood parameters (hemoglobin, thrombocytes, urea and creatinine) showed only weak correlations with CD55 or CD59 expression levels in the GI, HUS and HUS/N group (r ≤0.6, R^2^≤0.3; see figures S2–S4). The strongest significant correlation was found between hemoglobin and CD55 expression levels on erythrocytes in the GI and HUS groups (p = 0.05 and p = 0.009, respectively), but not in the HUS/N group (p = 0.27; see table 3). In leukocytes hemoglobin also yielded a significant correlation against CD55 expression levels in the GI group (p = 0.008), but not in the HUS and HUS/N groups (p = 0.276 and p = 0.737, respectively).

**Table 3:** Pearson-Bravais correlation tests between CD55 and CD59 expression and hemoglobin, thrombocytes, urea and creatinine levels of all patients included in the study.

Characteristic				Hemoglobin		Thrombocytes		Urea		Creatinine	
				CD55	CD59	CD55	CD59	CD55	CD59	CD55	CD59
	**GI** a	(n = 34)	r (r^2^)	0.33 (0.11)	0.56 (0.31)	0.06 (<0.01)	−0.20 (0.04)	−0.02 (<0.01)	−0.17 (0.03)	0.16 (0.03)	0.21 (0.04)
			p	0.054	0.001	0.731	0.255	0.899	0.344	0.370	0.235
**Erythro-cytes**	**HUS** a	(n = 23)	r (r^2^)	0.53 (0.28)	0.26 (0.07)	0.15 (0.02)	0.08 (0.01)	−0.03 (<0.01)	0.24 (0.06)	−0.08 (0.01)	0.16 (0.02)
			p	0.009	0.225	0.501	0.713	0.907	0.280	0.725	0.473
	**HUS/N** a	(n = 19)	r (r^2^)	−0.27 (0.07)	−0.29 (0.08)	0.25 (0.06)	0.45 (0.20)	−0.17 (0.03)	−0.32 (0.10)	0.00 (0.00)	−0.23 (0.05)
			p	0.266	0.234	0.313	0.056	0.477	0.180	0.992	0.350
	**GI** a	(n = 34)	r (r^2^)	−0.45 (0.20)	0.13 (0.02)	0.08 (0.01)	−0.06 (<0.01)	−0.10 (0.01)	0.05 (<0.01)	−0.24 (0.06)	0.03 (<0.01)
			p	0.008	0.467	0.635	0.725	0.565	0.779	0.177	0.889
**Leuko-cytes**	**HUS** a	(n = 23)	r (r^2^)	−0.24 (0.06)	0.04 (<0.01)	0.03 (<0.01)	0.03 (<0.01)	−0.33 (0.11)	−0.08 (0.01)	−0.29 (0.08)	−0.20 (0.04)
			p	0.276	0.869	0.880	0.904	0.120	0.734	0.186	0.369
	**HUS/N** a	(n = 19)	r (r^2^)	0.08 (0.01)	−0.51 (0.26)	−0.12 (0.01)	0.38 (0.15)	0.08 (0.01)	0.13 (0.02)	0.25 (0.06)	0.29 (0.08)
			P	0.737	0.027	0.639	0.105	0.738	0.591	0.311	0.229

a HC = healthy controls, GI = gastrointestinal symptoms, HUS = hemolytic uremic syndrome, HUS/N = HUS and neurological symptoms.

In summary, we discovered no lower, but a higher expression of CD55 and CD59 in patients after EAHEC infection, which were independent from clinical findings such as HUS development or neurological symptoms and did not correlate overall significantly with blood parameters like hemoglobin, thrombocytes, urea or creatinine.

### CD55 and CD59 Expression Levels in Patient Groups were Comparable Irrespectively of Previous Treatment with Plasma Separation and/or ECU

During the epidemic HUS patients were treated with plasma separation (PS) alone or PS and ECU in combination. We further examined the effect of PS and ECU on CD55 and CD59 expression on blood cells. HUS Patients were grouped according to their therapy into 4 groups with HUS patients who received plasma separation (n = 9), HUS patients who received plasma separation and ECU (n = 14), HUS/N patients who received plasma separation (n = 3) and HUS/N patients who received plasma separation and ECU (n = 16) as depicted in table 1.

**Figure 2:**
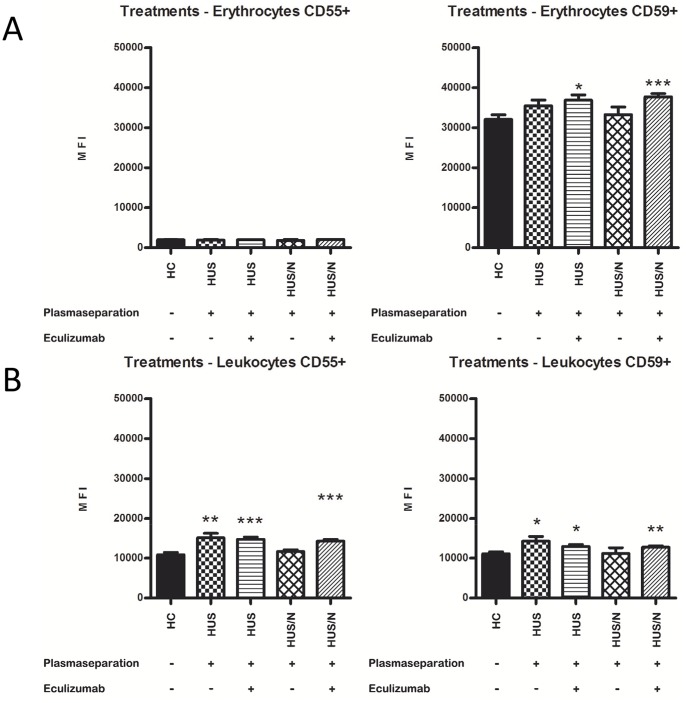
Plasma separation and ECU do not affect CD55 and CD59 expression on erythrocytes and leukocytes. Erythrocytes and leukocytes were incubated with CD45-, CD55- and CD59-specific antibodies and analyzed via flow cytometry. The CD45 marker was used to exclude or include the leukocyte population whereas the leukocyte subsets were distinguished via FSC/SSC-plot. HUS patients were grouped according to their therapy into 4 groups with HUS patients who received plasma separation (n = 9), HUS patients who received plasma separation and ECU (n = 14), HUS/N patients who received plasma separation (n = 3) and HUS/N patients who received plasma separation and ECU (n = 16). Healthy controls were also screened (HC, n = 12). A Erythrocytes, B Leukocytes. All values are given as mean fluorescence intensity (MFI) ± S.D. ANOVA, following symbols are used to pinpoint significant differences: vs. HC *. One symbols equals 0.05, two symbols 0.01, three symbols 0.001.

We found no differences in CD55 expression on erythrocytes between all four groups and HC (Fig. 2 A). The HUS and HUS/N groups treated with plasma separation and ECU showed a significantly higher expression of CD59 compared to HC (36835 and 37683 vs. 32068, p = 0.0152 and p = 0.0003 respectively). A trend towards a significantly higher CD59 expression was noticed in the HUS group treated with plasma separation compared to HC (35454 vs. 32068, p = 0.0793).

On leukocytes the CD55 and CD59 expression proved to be significantly higher for the HUS group treated with plasma separation and for both HUS and HUS/N groups treated with plasma separation and ECU compared to HC (Fig. 2 B; 15075, 14699 and 14255 vs. 10805, p = 0.0021, p = 0.0001 and p<0.0001 respectively for CD55; 14253, 12917 and 12705 vs. 11039, p = 0.0132, p = 0.0123 and p = 0.0060 respectively for CD59).

Taken together expression of CD55 was not different in ECU-treated and non-ECU treated patients, but CD59 expression was significantly higher in patients treated with PS and ECU compared to HC.

### Shiga toxin 2 had no Effect on CD55 and CD59 Expression Levels on Erythrocytes and Leukocytes *ex vivo*

Shiga toxin 2 represented the primary harmful virulence factor by EAHEC O104:H4 during infection. We studied the effect of Shiga toxin 2 on different blood cells of patients and HC in different concentrations for 24 h *ex vivo*. Afterwards CD55 and CD59 expression levels were assessed via flow cytometry. Patients were grouped according to their clinical course into 3 groups with GI (n = 4), HUS (n = 4) and HUS/N (n = 4).

**Figure 3:**
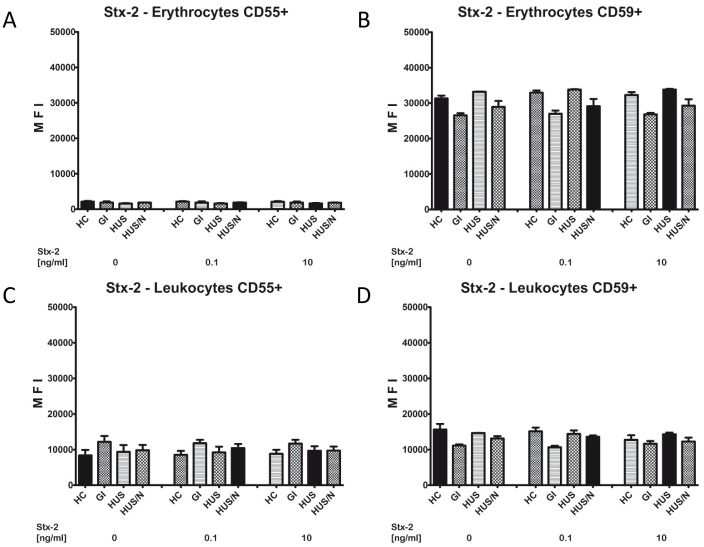
Stx-2 does not reduce the expression of CD55 and CD59 on erythrocytes and leukocytes. After incubating erythrocytes and leukocytes in whole blood with 0, 0.1 and 10/ml Shiga toxin 2 for 24 h the expression levels for CD55 and CD59 were analyzed via flow cytometry. Patients were grouped according to their clinical course into 3 groups with GI (n = 4), HUS (n = 4) and HUS/N (n = 4). Healthy controls were also screened (HC, n = 4) A CD55 expression, B CD59 expression on erythrocytes, C CD55 expression, D CD59 expression on leukocytes. All values are given as mean fluorescence intensity (MFI) ± S.D.

Adding Shiga toxin 2 had no effect on CD55 and CD59 expression on erythrocytes and leukocytes *ex vivo* from either healthy controls or former patients (Fig. 3 A–D).

## Discussion

In contrast to our initial hypothesis, CD55 and CD59 expression levels are not diminished neither in patients nor with HUS or HUS/N. Indeed, CD55 and CD59 expression were generally higher in subjects who recuperated from an infection with E. Coli O104:H4 than in HC. Thus CD55 and CD59 expression may not represent a suitable predictive marker for EAHEC patients at risk of HUS development.

In general, CD55 expression on erythrocytes is tightly regulated [8]. Indeed, mutations in the gene coding for CD55 are rare and even in patients with atypical HUS very limited [9], [10]. Alterations of CD55 protein have been reported in a malaria mouse model that leads to complement-mediated lysis of resistant erythrocytes [11]. A loss of CD55 expression on erythrocytes leads to anemia [12]–[16]. In our study 95% of the erythrocytes analyzed were positive for CD55 [data not shown].

CD59 is also known to protect cells from complement mediated lysis. A loss of CD59 function is causally involved in hemolytic anemia in PNH, HIV and malaria. [15], [17]–[22]. Interestingly, in our study the CD59 expression was higher in HUS patients and even higher in the HUS/N group indicating a role of CD59 expression on erythrocytes in compensating hemolytic anemia in HUS patients. A decrease in CD59 expression in asymptomatic hemolytic malaria patients and an increase in CD59 expression has been also shown in symptomatic hemolytic malaria patients compared to HC, underlining this hypothesis [12]. The group of Waitumbi, Opollo, Muga, Misore and Stoute showed that changes of ≤5–10% in CD59 expression on blood cells are correlated with clinical symptoms. This degree of change in CD59 expression is comparable to the results of our study. Similar findings have been reported for dengue hemorrhagic fever and the authors concluded that dysfunction in the complement system was in part responsible for an increase in hemolysis and tissue damage [23]. Hence, it seems that the hemolytic anemia caused by infection with E. Coli O104:H4 is being counteracted by a lasting elevation of CD59 on erythrocytes considering the mean erythrocyte life time of 120 days and the 3 month long time period since patients’ discharge. The mechanism behind this phenomenon still needs to be elucidated.

On leukocytes, granulocytes, monocytes and lymphocytes the expression of both, CD55 and CD59, was higher in the GI group compared to HC, and highest in the HUS group, but declined again in the HUS/N group. This effect may be explained by an insufficient increase in CD55 and CD59 in HUS patients evolving neurological symptoms (HUS/N). CD55 and CD59 function is not limited to the innate immune system by regulating the complement system. It has been shown that both CD55 and CD59 and other complement regulators are capable of attenuating T-cell responses and proliferation [24]–[29], which might explain the increased expression of CD55 and CD59 on leukocytes and their subsets according to disease severity and a possible involvement in HUS and HUS/N. A very interesting finding was recently published by Morigi, Galbusera, Gastoldi, Locatelli, Buelli et al. showing that Stx-2 induced on human microvascular endothelial cells the expression of P-selectin, which bound and activated complement via the alternative pathway [30]. Prior to this they had already demonstrated that Stx-2 was able to induce firm adhesion of human leukocytes to endothelium probably mediated by chemokines like CCL2/MCP-1 or IL8 [31]. These data deliver potential key elements for explaining HUS development in EAHEC patients. Unfortunately the finding regarding P-selectin was published towards the end of our study [30] so we could not include it as a parameter in our analysis in form of an endothelial cell study. This limitation should be taken care of in future EAHEC outbreaks and analysis of CD55 and CD59 expression extended by analysis of e.g. P- and L-selectin, LFA-1, MCP-1 and IL8.

Higher expression levels of CD55 and CD59 in patients after EAHEC infection (GI, HUS and HUS/N groups) did not correlate overall significantly with blood parameters like hemoglobin, thrombocytes, urea or creatinine. Yet, considering the time between admission and follow up/study inclusion it is imaginable that correlations during the acute early phase might be stronger. It would be interesting to investigate these early events in future outbreaks and to elucidate whether a change in one of these blood parameters is followed by an increase of CD55 and CD59 expression levels.

To our knowledge, a correlation between ECU treatment efficacy and CD55 and CD59 expression on erythrocytes and leukocytes has not been published so far. Neither the phase II clinical trials for ECU [32], nor the TRIUMPH phase III randomized study or even the SHEPHERD non-randomized safety and efficacy study [33], [34] tested for CD55 and CD59 expression on blood cells.

HUS or HUS/N patients treated with ECU displayed similar expression levels of CD55 and CD59 in comparison to patients not treated with ECU. The clinical outcome of ECU treatment for EAHEC O104:H4 dependent HUS was reported to be ineffective after metaanalysis of multiple patient collectives [35]. These findings and the results of our study do not support a significant role for CD55 and CD59 in HUS development during EAHEC O104:H4 infection and point to a different mechanism within the complement system for HUS development in EAHEC patients. A secondary phenomenon related to profound endothelial and platelet activation or injury might play a major role. Although constitutive expression of CD55 and CD59 on peripheral blood cells is stable, one major limitation of this study is that we were not able to analyze blood samples prospectively during the acute early phase. Future studies in similar outbreaks should look at these early events and include larger patient collectives in a multi-center study, if possible. Although our selected patients are highly representative, the total number of EAHEC infected patients in 2011 reached 3842 in northern Germany. Thus, a theoretical bias cannot be excluded since we conducted a single-center study without taking into account other medical centers.

CD55 and CD59 expression is higher on cells when challenged with bacteria or during inflammatory conditions [36]. However, we did not observe any effect of the bacterial toxin on CD55 and CD59 expression on erythrocytes or leukocytes within all patient groups and HCs. The toxic mechanisms of Shiga toxin 2 are not fully understood. It is reported, that Shiga toxin 2 is directly toxic to cells by inhibition of ribosome function. Recently, indirect toxic effects mediated by the complement system have been shown [30], [37], [38]. Shiga toxin 2 seems to inhibit the complement system protective factor H [37]. The results of our study are in agreement with the work of Orth, Khan, Naim, Grif, Brockmeyer et al. who also described that Shiga toxin 2 does not seem to interfere with CD55 or CD59 on blood cells although we cannot exclude functional effects on CD55 and CD59, because we only studied the phenotype of the blood cells.

We recommend with view on future EAHEC outbreaks longitudinal analysis of (1) CD46, CD55 and CD59 expression levels on erythrocytes and leukocytes, (2) serum markers of complement activation and protection (C3, C3a/C3b and other degradation products, factor B, factors C5 to C9 of the membrane attack complex MAC and degradation products thereof, factor H), (3) blood parameters of azotemia and anemia (hemoglobin, thrombocytes, urea and creatinine), (4) L-selectin and LFA-1 expression levels on leukocytes and (5) serum levels of CCL2/MCP-1 and IL8.

## Conclusions

In summary, we found HUS development in EAHEC O104:H4 infected patients to be independent from CD55 and CD59 expression on peripheral blood cells. Thus, our findings do not support a role for CD55 and CD59 in HUS development during EAHEC O104:H4 infection and point to a different mechanism within the complement system for HUS development in EAHEC patients, e.g. secondary phenomena related to profound endothelial and platelet activation or injury.

## Supporting Information

Figure S1 **Flow cytometric profiles for analysis of CD55 and CD59 expression on human erythrocytes and leukocytes.** Erythrocytes and leukocytes were incubated with CD45-, CD55- and CD59-specific antibodies. Graphs are representative of one patient. A, Erythrocytes were selected by using the CD45-specific Pacific Blue-channel to exclude all CD45+ leukocytes. Afterwards ranged gates were set to measure the CD55+ and CD59+ mean fluorescence intensity (MFI). B, Leukocytes and their subsets were selected by using the CD45-specific Pacific Blue channel and sideward scatter channel (SSC). The subsets were granulocytes, monocytes and lymphocytes. The same ranged gates were set as for erythrocytes.
(PDF)

Figure S2 **Pearson-Bravais correlation of CD55 and CD59 expression against blood parameters in the GI group.** Blood parameters for hemoglobin, thrombocytes, urea and creatinine were collected for all patients in the group with severe gastrointestinal symptoms (GI, n = 34). These values were correlated against the CD55 or CD59 expression levels. A Erythrocytes, B Leukocytes. Pearson-Bravais correlation results are given as r (correlation coefficient) and R^2^ (coefficient of determination).
(PDF)

Figure S3 **Pearson-Bravais correlation of CD55 and CD59 expression against blood parameters in the HUS group.** Blood parameters for hemoglobin, thrombocytes, urea and creatinine were collected for all patients in the group with HUS without neurological symptoms (HUS, n = 23). These values were correlated against the CD55 or CD59 expression levels. A Erythrocytes, B Leukocytes. Pearson-Bravais correlation results are given as r (correlation coefficient) and R^2^ (coefficient of determination).
(PDF)

Figure S4 **Pearson-Bravais correlation of CD55 and CD59 expression against blood parameters in the HUS/N group.** Blood parameters for hemoglobin, thrombocytes, urea and creatinine were collected for all patients in the group with HUS and neurological symptoms (HUS/N, n = 19). These values were correlated against the CD55 or CD59 expression levels. A Erythrocytes, B Leukocytes. Pearson-Bravais correlation results are given as r (correlation coefficient) and R^2^ (coefficient of determination).
(PDF)

Table S1 **Choice of isotype-controls and antibodies.**
(PDF)
