# Supplementary material for: Increased Expression of Complement Regulators CD55 and CD59 on Peripheral Blood Cells in Patients with EAHEC O104:H4 Infection
Source: PLoS One. 2013 Sep 23;8(9):e74880. doi: 10.1371/journal.pone.0074880 (PMC3781141; doi:10.1371/journal.pone.0074880)
Supplement: Figure S1 — Flow cytometric profiles for analysis of CD55 and CD59 expression on human erythrocytes and leukocytes. Erythrocytes and leukocytes were incubated with CD45-, CD55- and CD59-specific antibodies. Graphs are representative of one patient. A, Erythrocytes were selected by using the CD45-specific Pacific Blue-channel to exclude all CD45+ leukocytes. Afterwards ranged gates were set to measure the CD55+ and CD59+ mean fluorescence intensity (MFI). B, Leukocytes and their subsets were selected by using the CD45-specific Pacific Blue channel and sideward scatter channel (SSC). The subsets were granulocytes, monocytes and lymphocytes. The same ranged gates were set as for erythrocytes. (PDF) [file pone.0074880.s001.pdf]

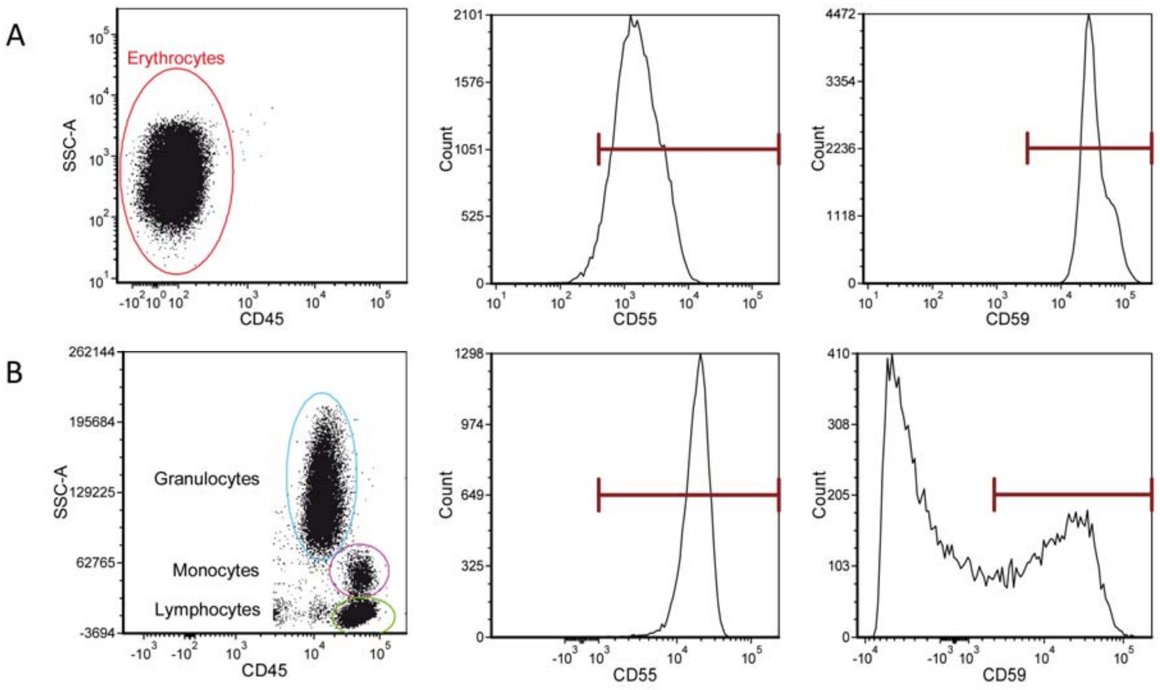

Supplementary figure 1 Flow cytometric profiles for analysis of CD55 and CD59 expression on human erythrocytes and leukocytes. Erythrocytes and leukocytes were incubated with CD45-, CD55- and CD59-specific antibodies. Graphs are representative of one patient. A, Erythrocytes were selected by using the CD45-specific Pacific Blue-channel to exclude all $CD45^+$ leukocytes. Afterwards ranged gates were set to measure the $CD55^+$ and $CD59^+$ mean fluorescence intensity (MFI). B, Leukocytes and their subsets were selected by using the CD45-specific Pacific Blue channel and sideward scatter channel (SSC). The subsets were granulocytes, monocytes and lymphocytes. The same ranged gates were set as for erythrocytes
