# Supplementary material for: Increased Expression of Complement Regulators CD55 and CD59 on Peripheral Blood Cells in Patients with EAHEC O104:H4 Infection
Source: PLoS One. 2013 Sep 23;8(9):e74880. doi: 10.1371/journal.pone.0074880 (PMC3781141; doi:10.1371/journal.pone.0074880)
Supplement: Figure S2 — Pearson-Bravais correlation of CD55 and CD59 expression against blood parameters in the GI group. Blood parameters for hemoglobin, thrombocytes, urea and creatinine were collected for all patients in the group with severe gastrointestinal symptoms (GI, n = 34). These values were correlated against the CD55 or CD59 expression levels. A Erythrocytes, B Leukocytes. Pearson-Bravais correlation results are given as r (correlation coefficient) and R2 (coefficient of determination). (PDF) [file pone.0074880.s002.pdf]

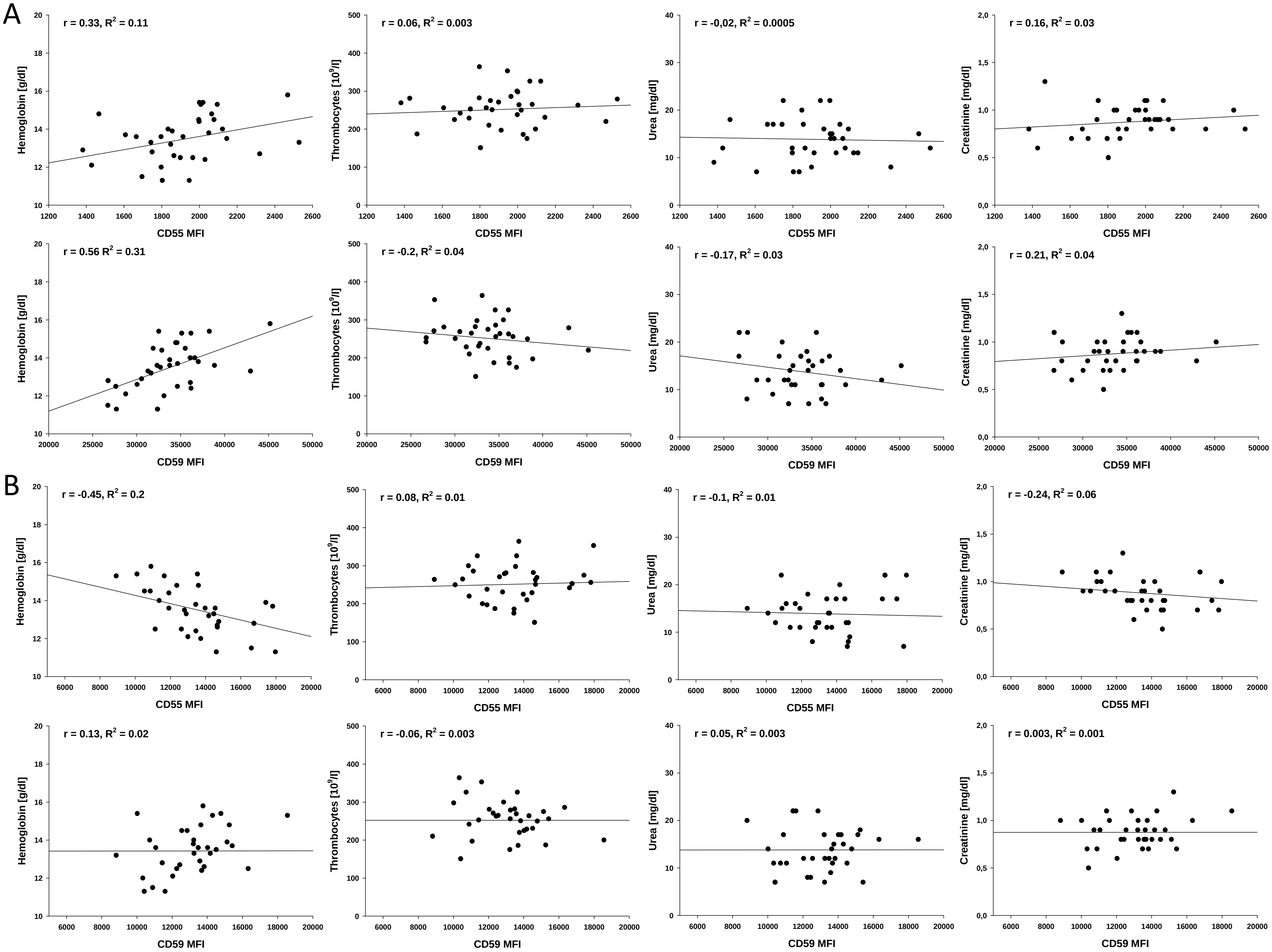
A
r = 0.33, R^2 = 0.11
Hemoglobin [g/dl]
CD55 MFI
r = 0.06, R^2 = 0.003
Thrombocytes [10^9/l]
CD55 MFI
r = -0,02, R^2 = 0.0005
Urea [mg/dl]
CD55 MFI
r = 0.16, R^2 = 0.03
Creatinine [mg/dl]
CD55 MFI
r = 0.56 R^2 = 0.31
Hemoglobin [g/dl]
CD59 MFI
r = -0.2, R^2 = 0.04
Thrombocytes [10^9/l]
CD59 MFI
r = -0.17, R^2 = 0.03
Urea [mg/dl]
CD59 MFI
r = 0.21, R^2 = 0.04
Creatinine [mg/dl]
CD59 MFI
B
r = -0.45, R^2 = 0.2
Hemoglobin [g/dl]
CD55 MFI
r = 0.08, R^2 = 0.01
Thrombocytes [10^9/l]
CD55 MFI
r = -0.1, R^2 = 0.01
Urea [mg/dl]
CD55 MFI
r = -0.24, R^2 = 0.06
Creatinine [mg/dl]
CD55 MFI
r = 0.13, R^2 = 0.02
Hemoglobin [g/dl]
CD59 MFI
r = -0.06, R^2 = 0.003
Thrombocytes [10^9/l]
CD59 MFI
r = 0.05, R^2 = 0.003
Urea [mg/dl]
CD59 MFI
r = 0.003, R^2 = 0.001
Creatinine [mg/dl]
CD59 MFI

Supplementary figure 2 Pearson-Bravais correlation of CD55 and CD59 expression against blood parameters in the GI group. Blood parameters for hemoglobin, thrombocytes, urea and creatinine were collected for all patients in the group with severe gastrointestinal symptoms (GI, n=34). These values were correlated against the CD55 or CD59 expression levels. A Erythrocytes, B Leukocytes. Pearson-Bravais correlation results are given as r (correlation coefficient) and R^2 (coefficient of determination).
