# Supplementary material for: Increased Expression of Complement Regulators CD55 and CD59 on Peripheral Blood Cells in Patients with EAHEC O104:H4 Infection
Source: PLoS One. 2013 Sep 23;8(9):e74880. doi: 10.1371/journal.pone.0074880 (PMC3781141; doi:10.1371/journal.pone.0074880)
Supplement: Figure S3 — Pearson-Bravais correlation of CD55 and CD59 expression against blood parameters in the HUS group. Blood parameters for hemoglobin, thrombocytes, urea and creatinine were collected for all patients in the group with HUS without neurological symptoms (HUS, n = 23). These values were correlated against the CD55 or CD59 expression levels. A Erythrocytes, B Leukocytes. Pearson-Bravais correlation results are given as r (correlation coefficient) and R2 (coefficient of determination). (PDF) [file pone.0074880.s003.pdf]

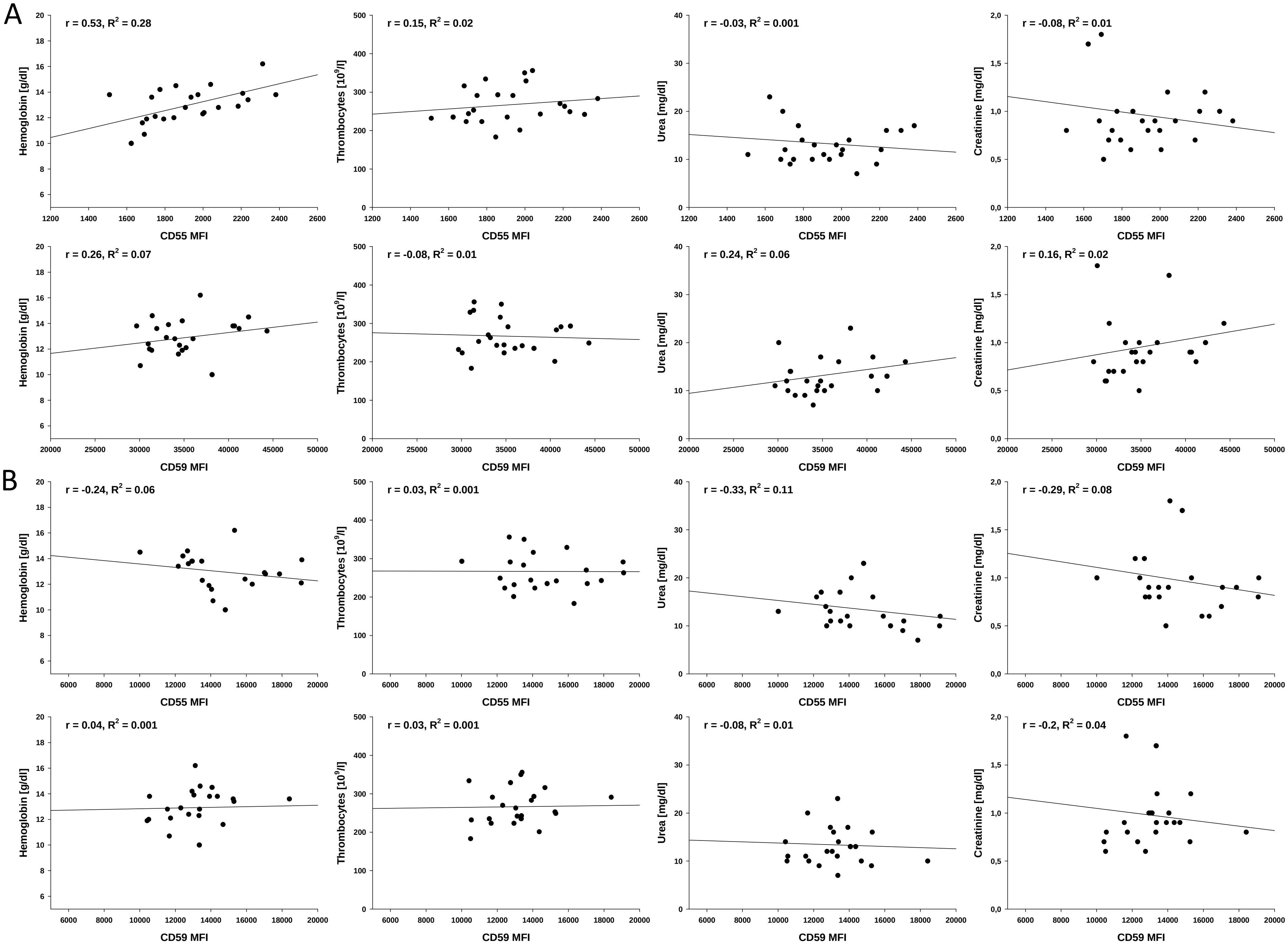

A
r = 0.53, R2 = 0.28
Hemoglobin [g/dl]
CD55 MFI
r = 0.15, R2 = 0.02
Thrombocytes [109/l]
CD55 MFI
r = -0.03, R2 = 0.001
Urea [mg/dl]
CD55 MFI
r = -0.08, R2 = 0.01
Creatinine [mg/dl]
CD55 MFI
r = 0.26, R2 = 0.07
Hemoglobin [g/dl]
CD59 MFI
r = -0.08, R2 = 0.01
Thrombocytes [109/l]
CD59 MFI
r = 0.24, R2 = 0.06
Urea [mg/dl]
CD59 MFI
r = 0.16, R2 = 0.02
Creatinine [mg/dl]
CD59 MFI
B
r = -0.24, R2 = 0.06
Hemoglobin [g/dl]
CD55 MFI
r = 0.03, R2 = 0.001
Thrombocytes [109/l]
CD55 MFI
r = -0.33, R2 = 0.11
Urea [mg/dl]
CD55 MFI
r = -0.29, R2 = 0.08
Creatinine [mg/dl]
CD55 MFI
r = 0.04, R2 = 0.001
Hemoglobin [g/dl]
CD59 MFI
r = 0.03, R2 = 0.001
Thrombocytes [109/l]
CD59 MFI
r = -0.08, R2 = 0.01
Urea [mg/dl]
CD59 MFI
r = -0.2, R2 = 0.04
Creatinine [mg/dl]
CD59 MFI

Supplementary figure 3 Pearson-Bravais correlation of CD55 and CD59 expression against blood parameters in the HUS group. Blood parameters for hemoglobin, thrombocytes, urea and creatinine were collected for all patients in the group with HUS without neurological symptoms (HUS, n=23). These values were correlated against the CD55 or CD59 expression levels. A Erythrocytes, B Leukocytes. Pearson-Bravais correlation results are given as r (correlation coefficient) and R^2 (coefficient of determination).
