# Supplementary material for: Increased Expression of Complement Regulators CD55 and CD59 on Peripheral Blood Cells in Patients with EAHEC O104:H4 Infection
Source: PLoS One. 2013 Sep 23;8(9):e74880. doi: 10.1371/journal.pone.0074880 (PMC3781141; doi:10.1371/journal.pone.0074880)
Supplement: Figure S4 — Pearson-Bravais correlation of CD55 and CD59 expression against blood parameters in the HUS/N group. Blood parameters for hemoglobin, thrombocytes, urea and creatinine were collected for all patients in the group with HUS and neurological symptoms (HUS/N, n = 19). These values were correlated against the CD55 or CD59 expression levels. A Erythrocytes, B Leukocytes. Pearson-Bravais correlation results are given as r (correlation coefficient) and R2 (coefficient of determination). (PDF) [file pone.0074880.s004.pdf]

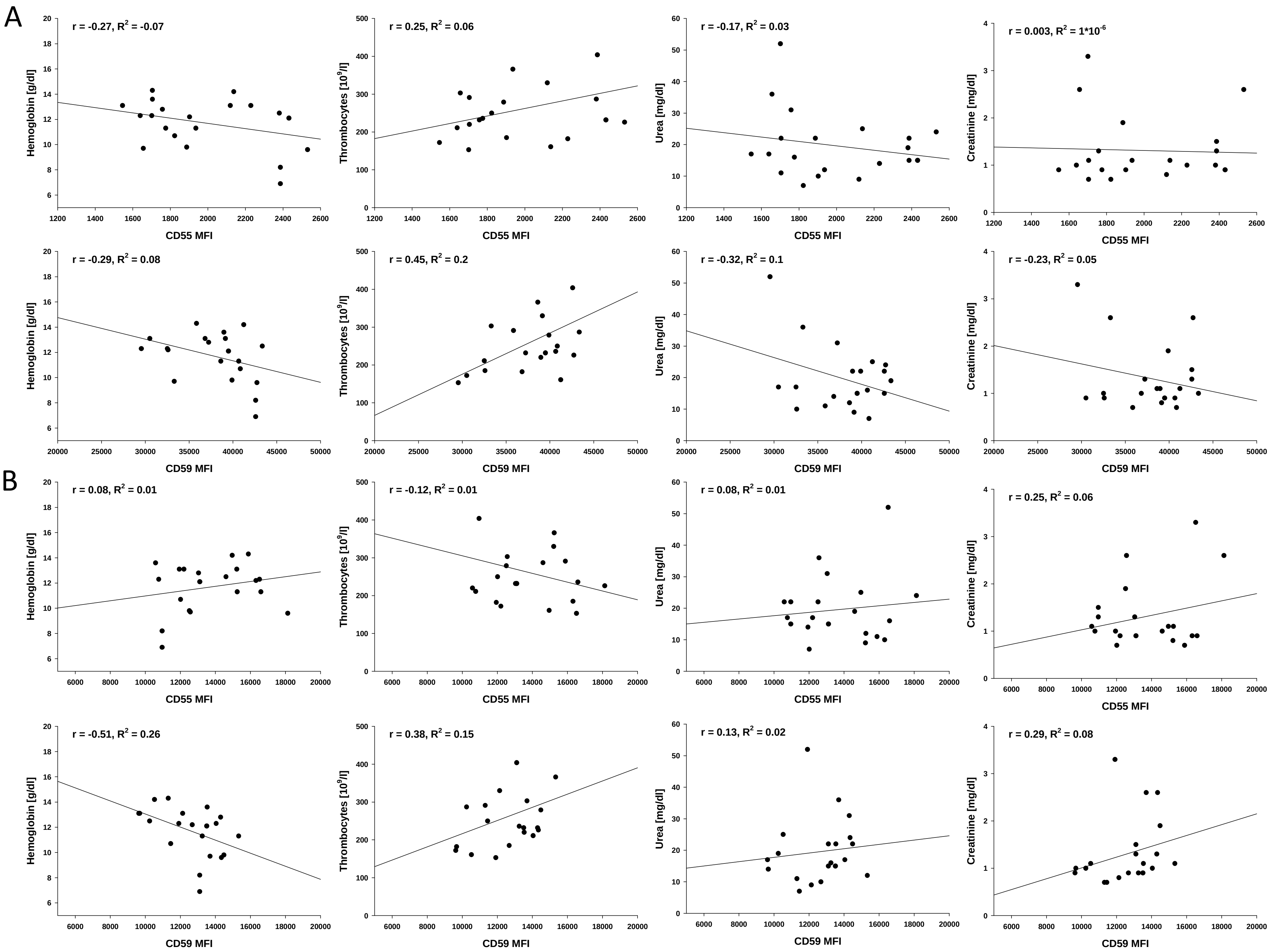

A
r = -0.27, R2 = -0.07
Hemoglobin [g/dl]
CD55 MFI
r = 0.25, R2 = 0.06
Thrombocytes [10^9/l]
CD55 MFI
r = -0.17, R2 = 0.03
Urea [mg/dl]
CD55 MFI
r = 0.003, R2 = 1*10^-6
Creatinine [mg/dl]
CD55 MFI
r = -0.29, R2 = 0.08
Hemoglobin [g/dl]
CD59 MFI
r = 0.45, R2 = 0.2
Thrombocytes [10^9/l]
CD59 MFI
r = -0.32, R2 = 0.1
Urea [mg/dl]
CD59 MFI
r = -0.23, R2 = 0.05
Creatinine [mg/dl]
CD59 MFI
B
r = 0.08, R2 = 0.01
Hemoglobin [g/dl]
CD55 MFI
r = -0.12, R2 = 0.01
Thrombocytes [10^9/l]
CD55 MFI
r = 0.08, R2 = 0.01
Urea [mg/dl]
CD55 MFI
r = 0.25, R2 = 0.06
Creatinine [mg/dl]
CD55 MFI
r = -0.51, R2 = 0.26
Hemoglobin [g/dl]
CD59 MFI
r = 0.38, R2 = 0.15
Thrombocytes [10^9/l]
CD59 MFI
r = 0.13, R2 = 0.02
Urea [mg/dl]
CD59 MFI
r = 0.29, R2 = 0.08
Creatinine [mg/dl]
CD59 MFI

Supplementary figure 4 Pearson-Bravais correlation of CD55 and CD59 expression against blood parameters in the HUS/N group. Blood parameters for hemoglobin, thrombocytes, urea and creatinine were collected for all patients in the group with HUS and neurological symptoms (HUS/N, n=19). These values were correlated against the CD55 or CD59 expression levels. A Erythrocytes, B Leukocytes. Pearson-Bravais correlation results are given as r (correlation coefficient) and R^2 (coefficient of determination).
