# Supplementary material for: Increased Expression of Complement Regulators CD55 and CD59 on Peripheral Blood Cells in Patients with EAHEC O104:H4 Infection
Source: PLoS One. 2013 Sep 23;8(9):e74880. doi: 10.1371/journal.pone.0074880 (PMC3781141; doi:10.1371/journal.pone.0074880)
Supplement: Table S1 — Choice of isotype-controls and antibodies. (PDF) [file pone.0074880.s005.pdf]

Supplementary table 1 Choice of isotype-controls and antibodies

Type	Company	Catalogue nr.	Fluorophore	Host	Isotype	Clone
**CD45 antibody, human**	Biolegend	304029	Pacific Blue	Mouse	IgG1, κ	HI30
**CD55 antibody, human**	Biolegend	311312	APC	Mouse	IgG1, κ	JS11
**CD59 antibody, human**	eBioscience	12-0596-42	PE	Mouse	IgG1, κ	OV92A2
**Isotype-control, human**	Biolegend	400151	Pacific Blue	Mouse	IgG1, κ	MOPC-21
**Isotype-control, human**	Biolegend	400120	APC	Mouse	IgG1, κ	MOPC-21
**Isotype-control, human**	Biolegend	400114	PE	Mouse	IgG1, κ	MOPC-21
